# An organic fluorophore-nanodiamond hybrid sensor for photostable imaging and orthogonal, on-demand biosensing

**DOI:** 10.1038/s41598-017-15772-0

**Published:** 2017-11-21

**Authors:** Malcolm S. Purdey, Patrick K. Capon, Benjamin J. Pullen, Philipp Reineck, Nisha Schwarz, Peter J. Psaltis, Stephen J. Nicholls, Brant C. Gibson, Andrew D. Abell

**Affiliations:** 1 ARC Centre of Excellence for Nanoscale BioPhotonics (CNBP), Adelaide, Australia; 2 Institute for Photonics and Advanced Sensing (IPAS), The University of Adelaide, North Terrace, Adelaide, SA 5005 Australia; 3 Department of Chemistry, School of Physical Sciences, The University of Adelaide, North Terrace, Adelaide, SA 5005 Australia; 4 South Australian Health and Medical Research Institute (SAHMRI) and School of Medicine, The University of Adelaide, North Terrace, Adelaide, SA 5001 Australia; 5 School of Science, RMIT University, Melbourne, VIC 3001 Australia

## Abstract

Organic fluorescent probes are widely used to detect key biomolecules; however, they often lack the photostability required for extended intracellular imaging. Here we report a new hybrid nanomaterial (peroxynanosensor, PNS), consisting of an organic fluorescent probe bound to a nanodiamond, that overcomes this limitation to allow concurrent and extended cell-based imaging of the nanodiamond and ratiometric detection of hydrogen peroxide. Far-red fluorescence of the nanodiamond offers continuous monitoring without photobleaching, while the green fluorescence of the organic fluorescent probe attached to the nanodiamond surface detects hydrogen peroxide on demand. PNS detects basal production of hydrogen peroxide within M1 polarised macrophages and does not affect macrophage growth during prolonged co-incubation. This nanosensor can be used for extended bio-imaging not previously possible with an organic fluorescent probe, and is spectrally compatible with both Hoechst 33342 and MitoTracker Orange stains for hyperspectral imaging.

## Introduction

**Figure 1:**
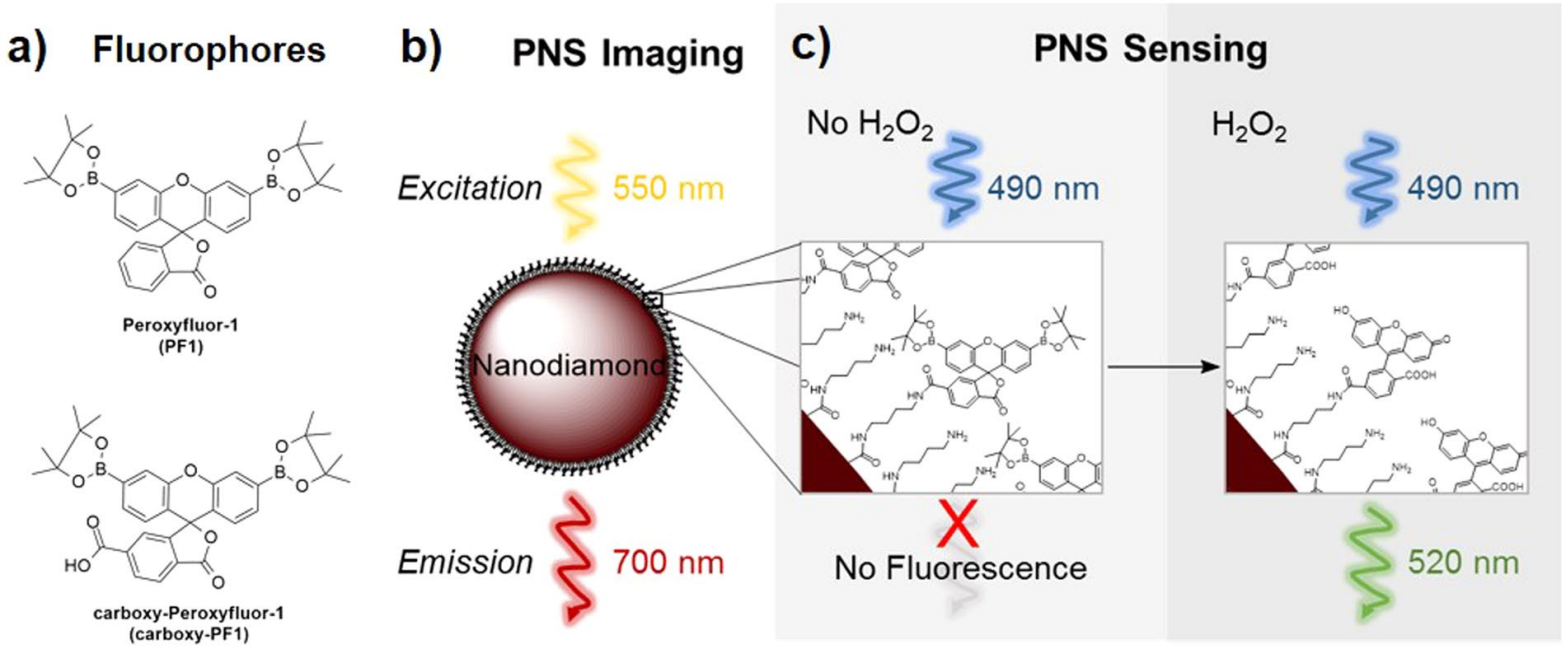
Peroxynanosensor (PNS) platform and fluorescence properties. (**a**) Structures of organic fluorophores PF1 and carboxy-PF1. (**b**) Scheme of PNS imaging. The nanodiamond is excited at 550 nm and emits stable fluorescence around 700 nm enabling long-term imaging. (**c**) Scheme of H_2_O_2_ sensing by PNS. The surface bound fluorophores (carboxy-PF1) are excited at 490 nm. In the absence of H_2_O_2_ it is mostly non-fluorescent and becomes highly fluorescent (520 nm) upon exposure to H_2_O_2_.

Biological markers such as hydrogen peroxide (H_2_O_2_)^1^, glutathione^2^, and metal ions^3^ play a central role in cell signalling and cellular development. These species are typically detected by measuring changes in fluorescence upon binding to or reacting with an organic fluorescent probe, e.g. peroxyfluor-1 (PF1, Fig. 1a). PF1 gives a large and measurable increase in green fluorescence on exposure to H_2_O_2_^4^. However, rapid photobleaching limits the use of such probes, with a concomitant reduction in fluorescence intensity over time. This is particularly problematic when using a high intensity light source for excitation of the fluorophore in optical fibre-based sensors^5^, and confocal microscopy^6^. This issue can, to some degree, be mitigated by decreasing excitation power or exposure time^7^, however the duration of the experiment and resolution of images collected is significantly limited by this approach. Synthetic derivatives can be prepared, but again any improvement in photostability is generally limited as even relatively photostable organic fluorophores are vulnerable to photobleaching under continuous illumination^8^. A general solution to this problem is needed, and here we present a new hybrid nanosensor approach. This consists of a photostable fluorescent nanomaterial (nanodiamonds, engineered to contain high concentrations of nitrogen-vacancy (NV) centres) for direct imaging and tracking in cells, and a surface-bound organic fluorescent probe for concurrent and orthogonal on-demand biosensing. This enables location of the hybrid sensor without direct optical excitation of the organic fluorophore, as the NV nanodiamond is excited instead. NV nanodiamonds are well suited to this purpose as they do not photobleach even under intense illumination^9^ and their fluorescence is mostly unchanged by interactions with biomolecules. This stability has been exploited for biological applications such as single molecule^10^ or cell^11^ tracking, bioconjugation for drug delivery^12^, study of intraneuronal transport abnormalities^13^, and *in vivo* tracking of macrophage cells injected with nanodiamonds^14^. Furthermore, NV nanodiamonds exhibit potential for small molecule sensing^15^ and Förster resonance energy transfer (FRET) with organic fluorophores^16,17^. Importantly, NV nanodiamonds also have low toxicity to cells and show good biocompatibility^12,18^.

The new nanosensor reported here consists of carboxy-PF1 molecules^19^ bound to the surface of an NV nanodiamond to yield a hybrid sensor (peroxynanosensor, referred to herein as PNS, see Fig. 1), which acts as a trackable, non-toxic, highly photostable nanosensor for H_2_O_2_. The NV nanodiamond is imaged in a cell by excitation at 550 nm and collection of the resultant fluorescence around 700 nm (Fig. 1b). Imaging the NV nanodiamond, rather than the organic carboxy-PF1 fluorophore, allows extended monitoring of PNS within a biological setting without any photobleaching of carboxy-PF1. Thus the surface bound carboxy-PF1 may be separately and orthogonally interrogated on demand (excitation 476 nm, emission 520 nm, Fig. 1c), which allows for long-term sensing of H_2_O_2_ that the equivalent organic fluorophore (PF1, Fig. 1a) is not capable of without significant photobleaching. The fluorescence intensity ratio between carboxy-PF1 and NV nanodiamond upon reaction of PNS with H_2_O_2_ provides a ratiometric measurement within a biological sample. Furthermore, PNS is spectrally compatible with the commonly used Hoechst 33342 and MitoTracker Orange stains for cell work, allowing for visualisation of cell nuclei and mitochondria concurrent with ratiometric H_2_O_2_ sensing.

## Results and Discussion

PNS was synthesised as outlined in Scheme S1 and detailed in the methods section. In brief, NV nanodiamonds with an average diameter of 120 nm were treated with H_2_SO_4_/HNO_3_ (9:1) to generate surface bound carboxylic acids^20^. Reaction with Fmoc-protected 1,4-diaminobutane linker in the presence of 1-[bis(dimethylamino)methylene]-1H-1,2,3-triazolo[4,5-b]pyridinium 3-oxid hexafluorophosphate (HATU) and diisopropylethylamine, and a subsequent Fmoc-deprotection, gave amine-functionalised nanodiamonds. These were coupled to carboxy-PF1 in the presence of *N*-hydroxysuccinimide and ethylcarbodiimide hydrochloride to give the nanosensor PNS.

**Figure 2:**
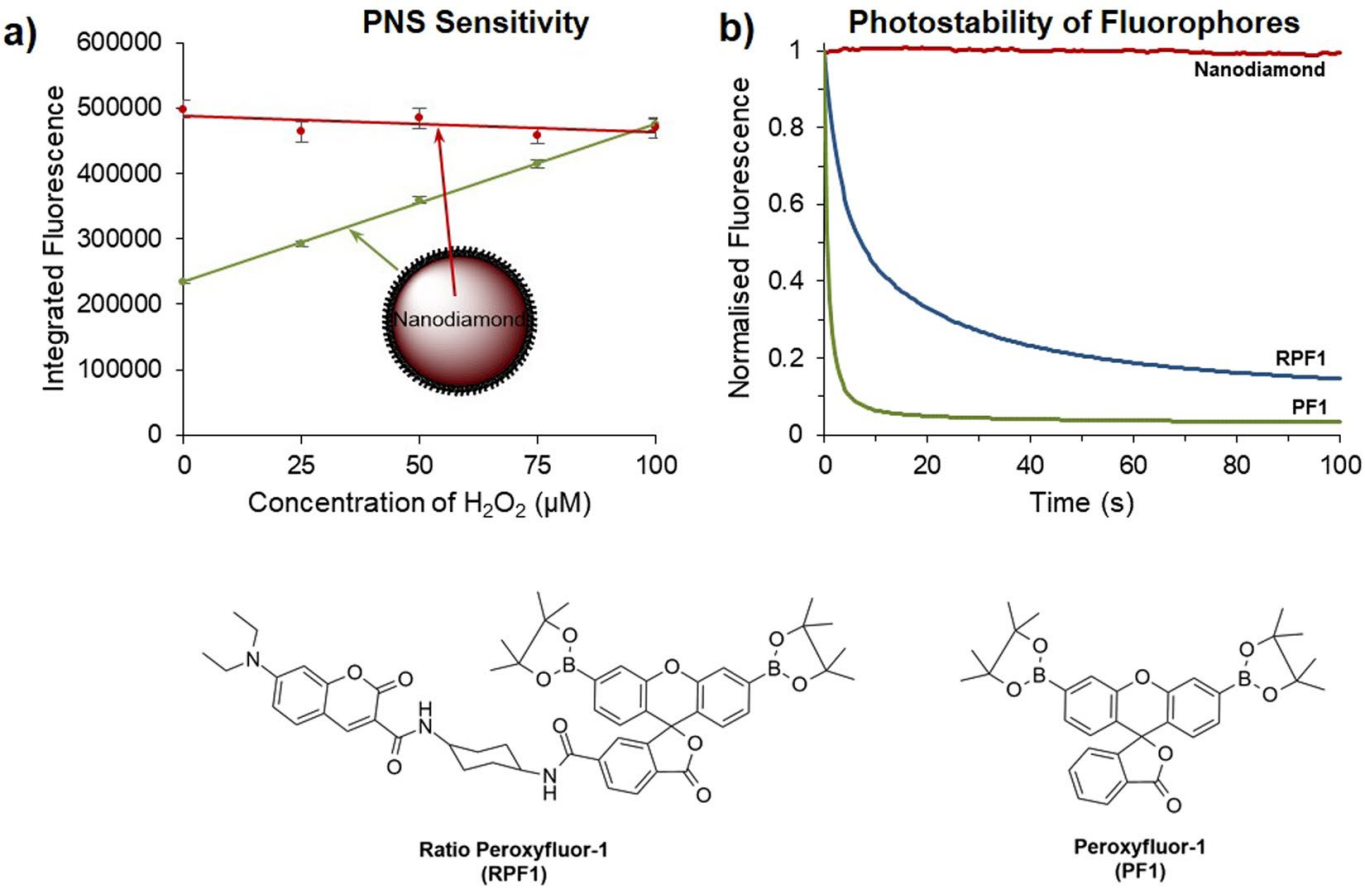
Sensitivity of PNS and photostability studies. (**a**) Dose-dependent response of PNS to H_2_O_2_ in solution. PNS was exposed to H_2_O_2_ in pH 7.5 phosphate buffered saline at 37 °C for 45 min. Integrated fluorescence intensity is shown at 510 nm–520 nm (carboxy-PF1, shown in green; excitation 450 nm) and at 690 nm–710 nm (NV nanodiamond, shown in red; excitation 550 nm). Each data point was collected in triplicate and averaged, and error bars show the standard deviation at each concentration. (**b**) Photobleaching of PF1 (green, excitation 485 nm, emission 520 nm), RPF1 (blue, excitation 400 nm, emission 520 nm) and NV nanodiamonds (red, excitation 560 nm, emission 700 nm). Excitation intensity was 12 Wcm^−2^, emission was normalised for each sample at t = 0 s and an image was recorded every 0.5 s.

The sensitivity of PNS to H_2_O_2_ in solution was assessed by incubation in 25–100 μM solutions of H_2_O_2_ for 45 min, with the resultant fluorescence response proportional to H_2_O_2_ concentration as shown in Fig. 2a. NV nanodiamond fluorescence remained essentially unchanged over the concentration range tested. A comparison of the two fluorescence peaks of PNS (520 nm from carboxy-PF1 and 700 nm from the NV nanodiamonds) provides ratiometric detection of H_2_O_2_, which contrasts simple fluorescence intensity-based detection of H_2_O_2_ using the equivalent organic fluorophore PF1 in isolation.

The photostability of the NV nanodiamonds was next compared to PF1, and also a second known ratiometric H_2_O_2_ sensor, ratioperoxyfluor-1 (RPF1; Fig. 2)^21^. Solutions of NV nanodiamonds (1 mg/mL), PF1 (100 μM) and RPF1 (100 μM) were treated with 10 equivalents of H_2_O_2_ for 3 h. Nanodiamond and organic fluorophore solutions were loaded into quartz capillaries that provided a well-defined sample geometry. Samples were illuminated using a wide-field fluorescence microscope at 12 W cm^−2^ (see Supplementary Fig. S1 and associated text for details) and imaged for 100 s, with the fluorescence intensity plotted as a function of time, see Fig. 2b. Photobleaching of PF1 is evident by a large reduction (>90%) in the fluorescence intensity after 10 s of irradiation. A smaller, but still significant, reduction in fluorescence (>50%) was observed for RPF1 in the first 10 s. Importantly, there was no evidence of photobleaching of the NV nanodiamond fluorescence after 100 s of irradiation, thus validating the use of the NV nanodiamond component to track PNS.

**Figure 3:**
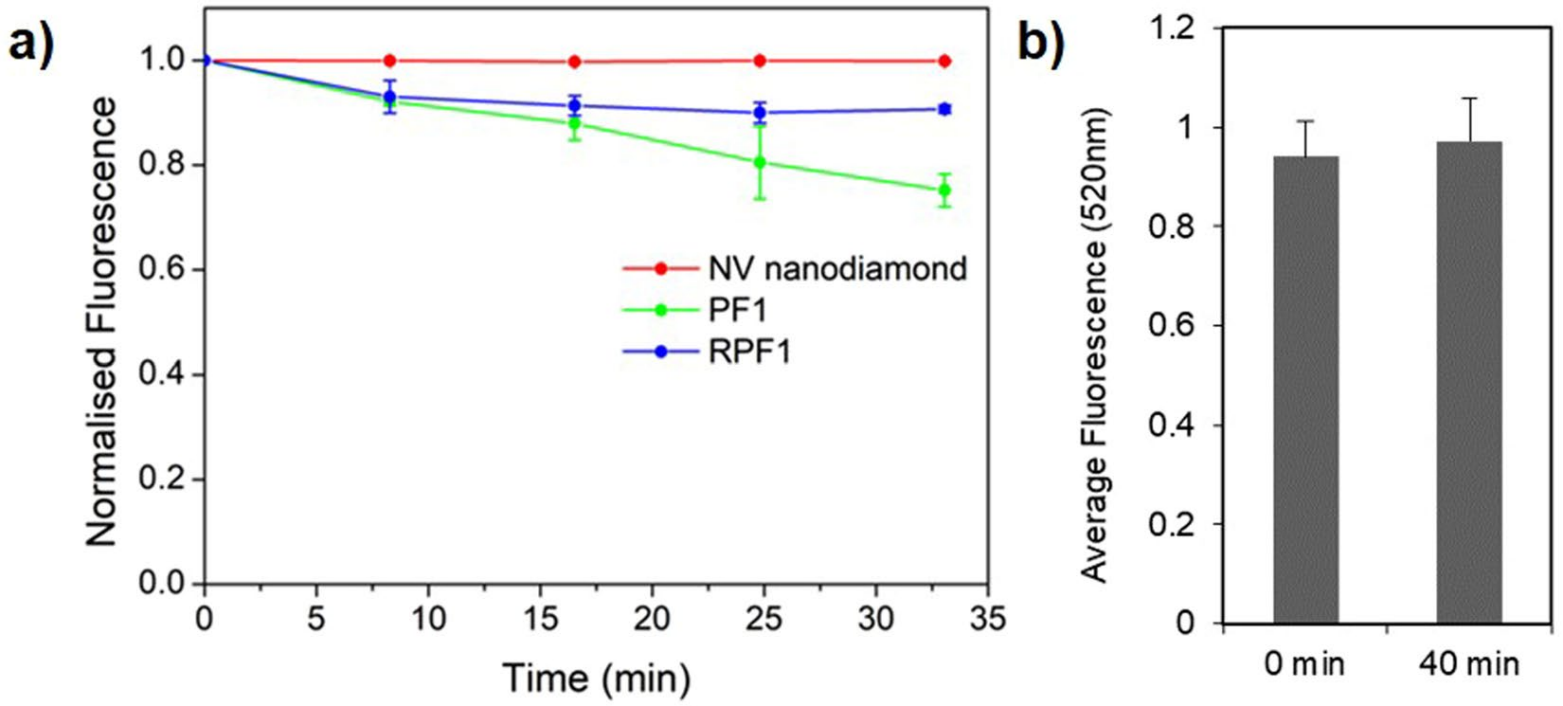
Photobleaching studies in macrophage cells by confocal microscopy. (**a**) Normalised fluorescence for PNS (red, NV nanodiamond excitation 560 nm, emission 700 nm), PF1 (green, excitation 485 nm, emission 520 nm), and RPF1 (blue, excitation 400 nm, emission 450 nm and 520 nm). An intensity reduction of 25% and 10% was observed for PF1 and RPF1 respectively, while no reduction occurred for the NV nanodiamond emission of PNS. (**b**) Fluorescence emission at 520 nm from surface bound carboxy-PF1 on PNS, which is unchanged over the 40 minutes of PNS imaging via the NV nanodiamond fluorescence. All error bars are the standard error of the mean.

The photostability of PNS in M1 polarised macrophages was compared to free organic fluorophores PF1 and RPF1 in order to investigate its utility in long-term microscopy experiments. Macrophages are associated with a range of diseases and produce high levels of reactive oxygen species, including H_2_O_2_, which is involved in macrophage phenotype regulation^22^. M1 polarised macrophages are typically associated with pro-inflammatory processes, and as such were used to better understand H_2_O_2_ signalling within the context of a pro-inflammatory disease state. Macrophages were obtained by harvesting the bone marrow from 12 week old C57BL/6 J mice and the bone marrow cells were differentiated to macrophages over 7 days. The culture media were supplemented with lipopolysaccharide and interferon-γ to polarise cells to the M1 phenotype. The macrophages were polarised for 48 h followed by incubation with one of PNS, PF1 or RPF1 for 30 min. The live cells were imaged in the confocal microscope five times over 30 min in order to measure any photobleaching of PNS, PF1, or RPF1 (Fig. 3a, see Supplementary Fig. S2 for cell images). Both PF1 and RPF1 showed a reduction in fluorescence intensity (25% and 10% respectively) over the course of imaging, while photobleaching was not observed for PNS. Furthermore, monitoring of PNS by the red NV nanodiamond emission does not photobleach the green emission of the surface bound carboxy-PF1 (Fig. 3b). This confirms that PNS may be imaged by the red NV nanodiamond fluorescence in macrophage cells without photobleaching the H_2_O_2_ sensing element, while the organic fluorophores PF1 and RPF1 are photobleached by imaging only 5 times in half an hour.

**Figure 4:**
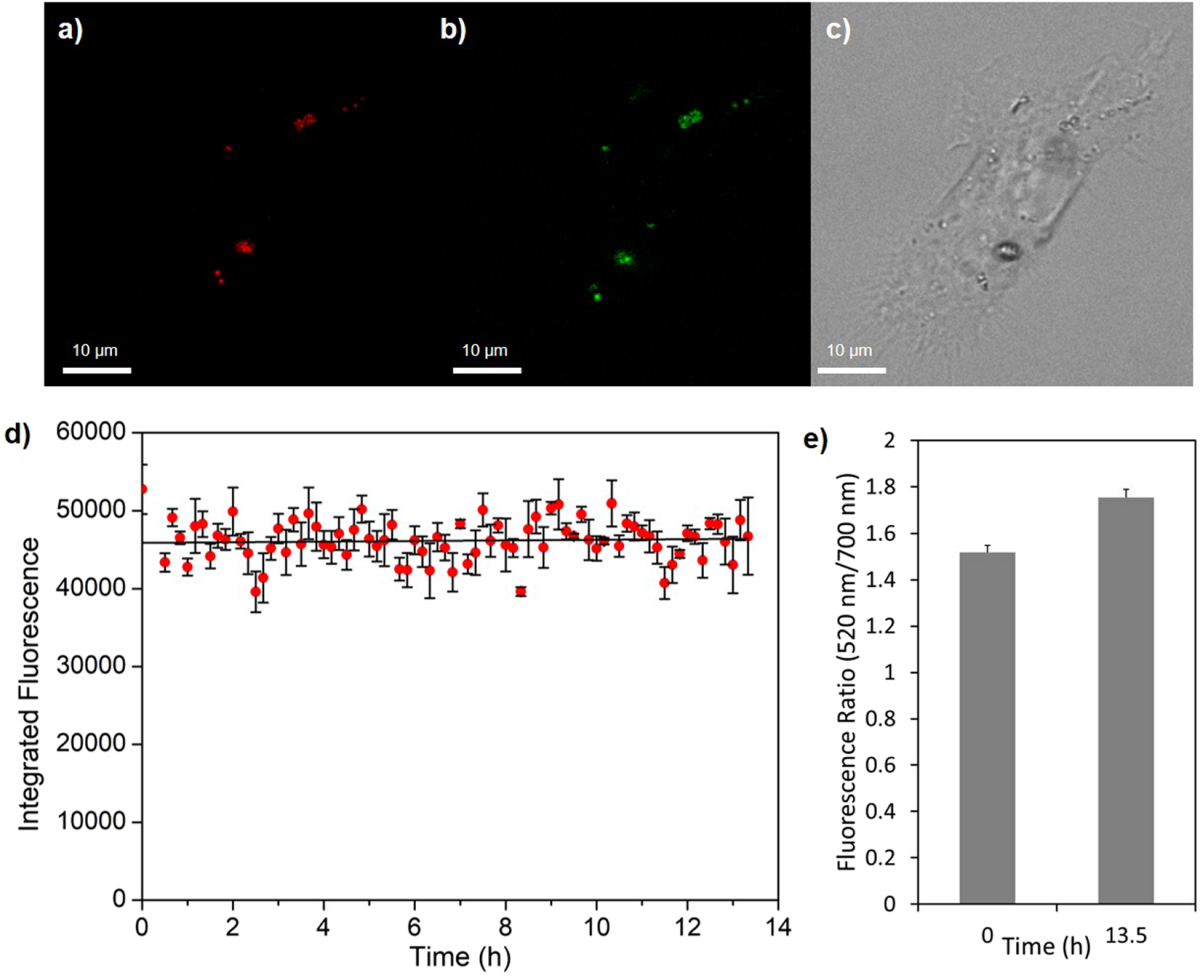
Long-term imaging of PNS by confocal microscopy. Images of a representative macrophage cell after 13.5 h showing (**a**) red emission (excitation 560 nm, emission 680–720 nm) of NV nanodiamond, (**b**) green emission (excitation 476 nm, emission 510–520 nm) of surface bound carboxy-PF1, and (**c**) brightfield contrast of the same cell. (**d**) Integrated fluorescence of the NV nanodiamond emission of PNS over 13.5 h of confocal microscopy (63x magnification, averaged results from three locations). (**e**) Fluorescence ratio of carboxy-PF1 to NV nanodiamond, which increased by 18% (p = 0.032) over the course of the 13.5 h experiment. All error bars are the standard error of the mean.

The ability of PNS to sense H_2_O_2_ was examined *in vitro* over an extended period. Macrophages were prepared as before, and after 35 h of polarisation the macrophages were incubated with PNS (10 µg/mL) for 1 h, washed, and imaged by confocal microscopy at 10 min intervals over the final 13.5 h of macrophage polarisation (see Fig. 4a–c for cell images). Surface bound carboxy-PF1 was imaged once by excitation at 476 nm, followed by monitoring of PNS in the macrophages over the 13.5 h experiment (NV nanodiamond excitation 560 nm, emission 680–720 nm). No photobleaching was apparent with the NV nanodiamond fluorescence effectively unchanged over the time course (Fig. 4d and Supplementary Video 1). At the end of the experiment, surface bound carboxy-PF1 was interrogated once more without evidence of photobleaching. In fact, the overall fluorescence ratio of the carboxy-PF1 compared to the NV nanodiamond (520 nm/700 nm) increased by 18% (p = 0.032), which reflects a basal level of H_2_O_2_ production by the macrophages over the last 13.5 h of polarisation (Fig. 4e). Thus it is clear that PNS is viable for long-term confocal microscopy experiments within macrophage cells, as H_2_O_2_ sensing is not affected by repeated imaging of the NV nanodiamond component. This allows for extended positional monitoring of PNS without an adverse impact on sensing capabilities.

Our new platform was used to measure H_2_O_2_ in macrophages above basal levels. PNS (10 µg/mL) was added to the culture media 17 h after initiation of macrophage polarisation. The cells were incubated with PNS for 1 h and then treated with an exogenous stimulus of 100 μM H_2_O_2_. Macrophage polarisation was completed (17 h) and the cells imaged by confocal microscopy. Cells initially treated with H_2_O_2_ showed a 50% higher PNS fluorescence ratio (comparison of 520 nm/700 nm) compared to untreated cells (see Supplementary Fig. S3; p = 0.041). PNS is thus capable of detecting higher levels of H_2_O_2_ while in macrophage cells and is able to report an increase in H_2_O_2_ 18 h after addition of the H_2_O_2_ solution to the culture media.

PNS was added to the culture media on day 2 of the differentiation process to assess any impact on macrophage growth. On day 7, the macrophage cells were polarised to the M1 phenotype as before for a further 2 days and then imaged by confocal microscopy (see Supplementary Fig. S4). Both the green and red fluorescence of PNS is visible within the macrophages after 7 days of co-incubation. Addition of PNS during the differentiation process did not affect macrophage growth, with typical morphology apparent. Hence, PNS is suitable for monitoring H_2_O_2_ production during cell proliferation and differentiation.

**Figure 5:**
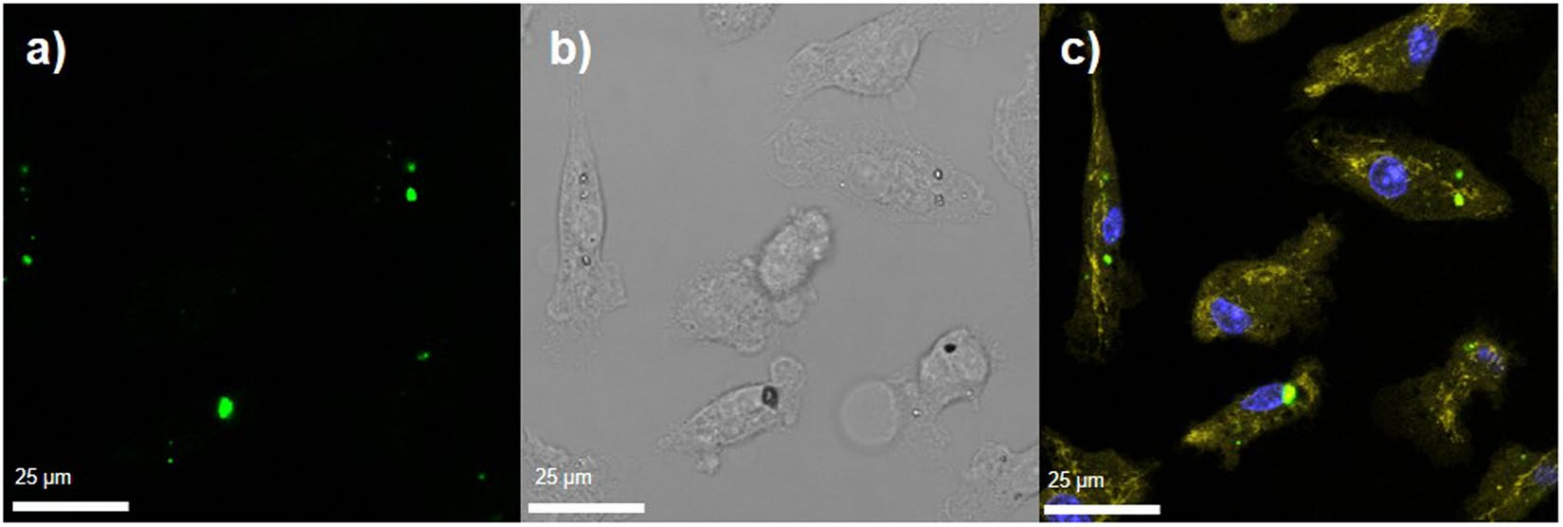
PNS multiplexed with Hoechst 33342 nuclear stain and MitoTracker Orange. The same cells are shown in all representative images and were collected using a confocal microscope under 63x magnification. (**a**) PNS green fluorescence, excitation 476 nm, emission 498–541 nm; (**b**) Brightfield contrast; (**c**) Merged image of all fluorescence channels; PNS green (as per A) and red fluorescence (excitation 554 nm, emission 719–770 nm); MitoTracker Orange (shown in yellow; excitation 554 nm, emission 571–578 nm) and Hoechst 33342 nuclear stain (shown as blue; excitation 405 nm, emission 460–480 nm). Note that PNS red fluorescence is masked by the PNS green fluorescence, which is significantly more intense.

Finally, the ability of PNS to be multiplexed with Hoechst 33342 nuclear stain and MitoTracker Orange was investigated. Hoechst 33342 and MitoTracker Orange are commonly used stains in biological experiments in order to visualise cell nuclei and mitochondria, respectively. It is desirable for PNS to be available for visualisation and H_2_O_2_ sensing without fluorescent signal overlap with these typical cellular stains. In this case, macrophages were incubated with PNS for 30 min and then incubated with MitoTracker Orange and Hoechst 33342 nuclear stain. The cells were fixed and imaged by confocal microscopy (Fig. 5 and Supplementary Video 2), with a clearly identifiable nucleus (indicated by blue Hoechst 33342) and mitochondria (indicated by MitoTracker Orange). Figure 5c demonstrates that PNS is not located within the cell nucleus and the fluorescent signals of surface bound carboxy-PF1 (green) and NV nanodiamond (red) are clearly distinguished from the fluorescent signals of Hoechst 33342 and MitoTracker Orange. Thus, PNS can be successfully multiplexed with other fluorophores for hyperspectral imaging, and cellular detail can be observed concurrent with H_2_O_2_ sensing.

In conclusion, attachment of an organic fluorescent probe (carboxy-PF1) to an NV nanodiamond provided a new hybrid nanomaterial (PNS) capable of extended imaging and detection of H_2_O_2_, without photobleaching normally associated with imaging the separate organic fluorophore alone. The NV nanodiamond component can be continuously visualised over an extended period, while the attached carboxy-PF1 is separately interrogated when measurement of H_2_O_2_ is desired. PNS detects basal production of H_2_O_2_ by M1 polarised macrophages, as well as exogenously introduced H_2_O_2_. This process allows for ratiometric measurement of H_2_O_2_ levels by comparison of the NV nanodiamond and carboxy-PF1 fluorescence. Incubation of PNS in macrophages during the final 5 days of differentiation and 2 days of polarisation does not affect cell morphology. In addition, the new sensing platform is spectrally compatible with standard fluorophores Hoechst 33342 and MitoTracker Orange. The high photostability, compatibility with existing fluorescent stains, and ability for on-demand ratiometric detection of H_2_O_2_ during extended cell based imaging presents PNS as an excellent new tool for imaging and biosensing.The approach presented here should be generally applicable to other fluorophores.

## Methods

All chemicals were purchased from Sigma-Aldrich unless otherwise stated. N-Fmoc-1,4-butanediamine was purchased from ChemImpex (Wood Dale, IL, USA). 100 mM Phosphate buffer solutions were prepared from monosodium phosphate and disodium phosphate in Milli-Q water. Nanodiamond particles were purchased from Nabond, Hong Kong. Ultrasonication of NV nanodiamonds was performed using a FS-600N ultrasonicator homogeniser from Zhengzhou HengChen Electronic (Zhengzhou, HE, China) fitted with a 3 mm probe. All confocal microscopy was performed on a Leica Microsystems TCS SP8X/MP confocal microscope (Wetzlar, Germany). MitoTracker® Orange CMTMRos and Hoechst 33342 solution (20 mM) was purchased from ThermoFisher Scientific. Fourier Transform Infrared Spectroscopy (FTIR) was performed using a Perkin Elmer S400 Infrared spectrometer in Universal ATR mode.

### Synthesis of PNS

Nanodiamond particles were irradiated with high-energy electrons (2 MeV) to a total fluence of 1 × 10^18^ cm^−2^. The particles were annealed in vacuum for 2 h at 800 °C and subsequently oxidised in air at 510 °C for 4.5 h at an air flow rate of 14 mL/min. NV nanodiamonds were suspended in Milli-Q water (10 mL) by ultrasonication for 1 h to a concentration of 1 mg/mL. The nanodiamonds were pelleted by centrifugation at 7800 rpm for 20 min and the supernatant removed. The nanodiamonds were resuspended in a 9:1 mixture of H_2_SO_4_:HNO_3_ (10 mL) and stirred at 70 °C for 18 h. The suspension was diluted with water (10 mL), before the carboxylate functionalised nanodiamonds were pelleted (as above) and subsequently resuspended in water (2 × 10 mL). The nanodiamonds were pelleted and resuspended in 0.1 M sodium hydroxide solution (10 mL) and stirred at 90 °C for 1 h. The solution was cooled to rt, the nanodiamonds were pelleted and resuspended in water (2 × 10 mL) then 0.1 M hydrochloric acid (10 mL). This acidic suspension of nanodiamonds was stirred at 90 °C for 1 h and pelleted and resuspended in water (3 × 10 mL) followed by dimethyl formamide (DMF, 2 × 10 mL). The nanodiamond suspension was pelleted and resuspended in a solution of 1-[bis(dimethylamino)methylene]-1H-1,2,3-triazolo[4,5-b]pyridinium 3-oxid hexafluorophosphate (HATU, 380.2 mg, 1 mmol) and diisopropylethylamine (348 μL, 2 mmol) in DMF (10 mL), then ultrasonicated for 20 min. N-Fmoc-1,4-butanediamine (173 mg, 0.5 mmol) was added to the suspension and the reaction mixture ultrasonicated for 1 h. The functionalised nanodiamonds were pelleted and resuspended in DMF (2 × 10 mL), then resuspended in a 20% solution of piperidine in DMF (10 mL). This suspension of amino functionalised nanodiamonds was ultrasonicated for 1 h, pelleted and resuspended in DMF (2 × 10 mL). Meanwhile, a solution of carboxy-PF1 (4 mg, 7 µmol), *N*-hydroxysuccinimide (28 mg, 0.25 mmol) and ethylcarbodiimide hydrochloride (48 mg, 0.25 mmol) in DMF (1 mL) was stirred for 20 min. This solution was added to the nanodiamond suspension, and the resultant mixture was ultrasonicated for 20 min. The functionalised nanodiamonds were pelleted and resuspended in DMF (2 × 10 mL) and water (2 × 10 mL) then lyophilised to give PNS as a white powder.

Attachment of the diamine linker was confirmed by FTIR, with characteristic signals for C-H stretching (2930 cm^−1^) and C-H bending (1453 cm^−1^) modes observed for the amino functionalised NV nanodiamond- intermediate. Prior to functionalisation, a broad O-H stretch (~3400 cm^−1^) is observed in the NV nanodiamond FTIR spectrum. The loss of this O-H stretch upon attachment of the butane linker provides further evidence for successful attachment to the NV nanodiamond. The characteristic C-H signals from the butane linker were retained in the FTIR spectrum of PNS, along with an additional stretch at 807 cm^−1^. The additional stretch indicates the presence of a boronate ester^23^, as expected due to the two boronate esters in carboxy-PF1 prior to reaction with H_2_O_2_ (see Fig. 1a for carboxy-PF1 structure and see Supplementary Fig. S5 for full FTIR spectra).

### Photostability of PNS, PF1 and RPF1 in capillary tubes

Photostability experiments were performed as previously reported^8^. Fluorescence images were obtained using a Olympus IX83 frame, a 40 × (NA 0.95) objective (SP-40X/UPLSAPO-100XO, Olympus, Japan), fitted with a Hamamatsu ORCA-Flash4.0 digital CMOS camera (Hamamatsu Photonics, Japan). A SPECTRA X light engine (Lumencor Inc., USA) was used for excitation and the power calibrated with a PM100D power meter. Glass capillaries (No. 5001-050, VitroCom, USA) were used as received, brought into contact with the PNS, PF1 or RPF1 solution, and the solution was allowed to fill the capillary via capillary forces. Average fluorescence intensities IS and IB inside the capillaries were determined using ImageJ^24^. The fluorescence intensity was averaged for bleaching experiments within an area of ca. 35 μm^2^ in the centre of the capillary (in order to minimise the effect of diffusion on the photobleaching dynamics), and it was ensured that diffusion from unilluminated areas of the capillary into the field of view did not affect the measurement by calculating the mean square displacement due to Brownian motion based on particle size.

### Macrophage collection and differentiation

Bone marrows were harvested from 12 week old C57BL/6J mice and approximately 1 × 10^6^/mL cells were seeded in 4-well ibiTreat µ-Slide chamber slides (Ibidi; Martinsried, Germany). The bone marrow cells were differentiated to macrophages in RPMI-1640 media, supplemented with 10 ng/mL M-CSF, 10% foetal bovine serum and 1% antibiotic-antimycotic, for 7 days and media was changed every three days. On day 7, the culture media were supplemented with 25 ng/mL lipopolysaccharide from *E.coli* (LPS) and 5 ng/mL interferon-γ to polarise cells to the M1 phenotype. The macrophages were polarised for 48 h followed by incubations with PNS or organic fluorophore as desired.

### Photostability of PNS, PF1 and RPF1 in macrophage cells

Macrophages were collected and differentiated as above, and then incubated with either PNS (10 µg/mL), PF1 (10 μM) or RPF1 (10 μM) for 1 h. The cells were washed, then imaged at 37 °C and 6% CO_2_ by confocal microscopy. PNS was excited at 476 nm and 560 nm; emission was collected from 510–520 nm and 670–732 nm. PF1 was excited at 476 nm and emission collected from 510–520 nm. RPF1 was excited at 405 nm and emission collected from 455–479 nm and 510–524 nm. Each well was imaged at 40x magnification in three different locations across the well and these results averaged. Each location was imaged five times across 30 min. The averaged fluorescence intensity of a treatment over time was then normalised to its initial average fluorescence value. This allowed for comparison between PNS, PF1 and RPF1.

### Confocal Imaging PNS in macrophages over 13.5 h

Macrophages were collected and differentiated as above. Samples were incubated with PNS (10 µg/mL) for 1 h, before being washed. The cells were then imaged by confocal microscopy at 37 °C and 6% CO_2_ under 63x magnification at three different representative locations. Z-stack images were taken, collecting fluorescence at both 520 nm and 700 nm (excitation 476 and 560 nm respectively). The cells were then imaged continually over 13.5 h, collecting only the red nanodiamond fluorescence at 700 nm (560 nm excitation only). Finally, the cells were imaged again, collecting both 520 nm and 700 nm fluorescence (excitation 476 and 560 nm respectively). The images were processed by ImageJ, removing background autofluorescence to give the fluorescence intensity from three consecutive z slices in the centre of the cell at each location. The ratio of 520 nm/700 nm fluorescence was then calculated from these intensities.

### Confocal Imaging response of PNS to macrophages treated with H_2_O_2_

Macrophages were collected and differentiated as above. Two samples were incubated with PNS (10 µg/mL) for 1 h, before being washed. The macrophages were then incubated in media for a further 18 h in either control media, or with added H_2_O_2_ (100 μM). The cells were then imaged by confocal microscopy at room temperature under 20x magnification at four different representative locations for each condition. The images were processed by ImageJ, removing background autofluorescence to give the fluorescence intensity from three consecutive z slices in the centre of the cell at each location. The ratio of 520 nm/700 nm fluorescence was then calculated from these intensities.

### Stability and toxicity of PNS in macrophages cells

Macrophages were collected and differentiated as above. On day 2 of the differentiation, PNS (10 µg/mL) was added, incubated for 1 h and then washed. On day 7, the culture media were supplemented with 25 ng/mL lipopolysaccharide from E.coli and 5 ng/mL interferon-γ to polarise cells to the M1 phenotype. After a further 48 h, the cells were imaged by confocal microscopy, a total of 7 days after the addition of PNS. Confocal settings for excitation and emission were as for H_2_O_2_ sensing experiments above.

### Data availability

The datasets generated during and/or analysed during the current study are available from the corresponding author on reasonable request.

## Electronic supplementary material

Supplementary video 1

Supplementary video 2

Supporting Information

## References

[1] Lippert AR, Van de Bittner GC, Chang CJ (2011). Boronate oxidation as a bioorthogonal reaction approach for studying the chemistry of hydrogen peroxide in living systems. Acc. Chem. Res..

[2] Niu LY (2012). BODIPY-based ratiometric fluorescent sensor for highly selective detection of glutathione over cysteine and homocysteine. J. Am. Chem. Soc..

[3] Carter KP, Young AM, Palmer AE (2014). Fluorescent sensors for measuring metal ions in living systems. Chem. Rev..

[4] Chang MCY, Pralle A, Isacoff EY, Chang CJ (2004). A selective, cell-permeable optical probe for hydrogen peroxide in living cells. J. Am. Chem. Soc..

[5] Purdey MS, Thompson JG, Monro TM, Abell AD, Schartner EP (2015). A dual sensor for pH and hydrogen peroxide using polymer-coated optical fibre tips. Sensors.

[6] Bernas T, Zarebski M, Cook RR, Dobrucki JW (2004). Minimizing photobleaching during confocal microscopy of fluorescent probes bound to chromatin: role of anoxia and photon flux. J. Microsc..

[7] Hoebe RA (2007). Controlled light-exposure microscopy reduces photobleaching and phototoxicity in fluorescence live-cell imaging. Nat. Biotechnol..

[8] Reineck P (2016). Brightness and photostability of emerging red and near-IR fluorescent nanomaterials for bioimaging. Adv. Opt. Mater..

[9] Yu S-J, Kang M-W, Chang H-C, Chem K-M, Yu Y-C (2005). Bright fluorescent nanodiamonds: no photobleaching and low cytotoxicity. J. Am. Chem. Soc..

[10] Hui YY (2017). Single particle tracking of fluorescent nanodiamonds in cells and organisms. Curr. Opin. Solid State Mater. Sci..

[11] Su LJ (2017). Fluorescent nanodiamonds enable quantitative tracking of human mesenchymal stem cells in miniature pigs. Sci. Rep..

[12] Zhu Y (2012). The biocompatibility of nanodiamonds and their application in drug delivery systems. Theranostics.

[13] Haziza S (2016). Fluorescent nanodiamond tracking reveals intraneuronal transport abnormalities induced by brain-disease-related genetic risk factors. Nat. Nanotechnol..

[14] Melzer S, Ankri R, Fixler D, Tarnok A (2015). Nanoparticle uptake by macrophages in vulnerable plaques for atherosclerosis diagnosis. J. Biophotonics.

[15] Liu B (2016). Application of nanodiamonds in Cu(ii)-based rhodamine B probes for NO detection and cell imaging. J. Mater. Chem. B.

[16] Tisler J (2011). Highly efficient FRET from a single nitrogen-vacancy center in nanodiamonds to a single organic molecule. ACS Nano.

[17] Fudala R (2014). FRET enhanced fluorescent nanodiamonds. Curr. Pharm. Biotechnol..

[18] Huang YA (2014). The effect of fluorescent nanodiamonds on neuronal survival and morphogenesis. Sci. Rep..

[19] Purdey MS (2015). Boronate probes for the detection of hydrogen peroxide release from human spermatozoa. Free Radical Biol. Med..

[20] Krueger A, Lang D (2012). Functionality is key: recent progress in the surface modification of nanodiamond. Adv. Funct. Mater..

[21] Albers AE, Okreglak VS, Chang CJ (2006). A FRET-based approach to ratiometric fluorescence detection of hydrogen peroxide. J. Am. Chem. Soc..

[22] He, C. & Carter, A. B. The metabolic prospective and redox regulation of macrophage polarization. *J. Clin. Cell. Immunol*. **6**, 10.4172/2155-9899.1000371 (2015). 10.4172/2155-9899.1000371 PMC4780841 26962470

[23] Butcher FK, Gerrard W, Howarth M, Mooney EF, Willis HA (1964). The infrared spectra of the aryl boronate esters derived from catechol and 2: 3 dihydroxynapthalene. Spectrochim. Acta.

[24] Schneider CA, Rasband WS, Eliceiri KW (2012). NIH Image to ImageJ: 25 years of image analysis. Nat. Methods.

