# Supplementary material for: An organic fluorophore-nanodiamond hybrid sensor for photostable imaging and orthogonal, on-demand biosensing
Source: Sci Rep. 2017 Nov 21;7:15967. doi: 10.1038/s41598-017-15772-0 (PMC5698319; doi:10.1038/s41598-017-15772-0)
Supplement: Supplementary file 3 — Supporting Information [file 41598_2017_15772_MOESM3_ESM.pdf]

Supporting Information

An organic fluorophore-nanodiamond hybrid sensor for photostable imaging and orthogonal, on-demand biosensing

Malcolm S. Purdey, Patrick K. Capon, Benjamin J. Pullen, Philipp Reineck, Nisha Schwarz, Peter J. Psaltis, Stephen J. Nicholls, Brant C. Gibson and Andrew D. Abell

Contents

1. Experimental 1
   1.1. Synthesis of PF1, carboxy-PF1 and RPF1 1
2. Supplementary Figures 3
   2.1. Brightness comparison of PNS, PF1 and RPF1 3
   2.2. Bio-photostability of PNS compared to PF1 and RPF1 4
   2.3. Detection of H_2O_2 as an exogenous stimulus 6
   2.4. PNS in Macrophages after 7 days 7
   2.5. Fourier-Transform Infrared Spectroscopy 7
3. References 8

1. Experimental

1.1. Synthesis of PF1, carboxy-PF1 and RPF1

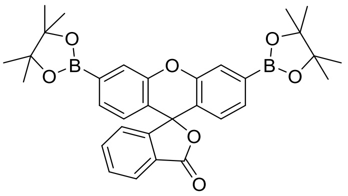

PF1 was prepared as per previous protocols.[1] Diiodofluoran[2] (600 mg, 1.1 mmol), bis(pinacolato)diboron (830 mg, 3.3 mmol), potassium acetate (640 mg, 6.5 mmol) and Pd(dppf)Cl_2 (80 mg, 0.11 mmol) were dried under high vacuum overnight. The reaction vessel was sealed, dry dimethylformamide (DMF, 10 mL) was added and argon gas bubbled through. The reaction mixture was stirred at 80 °C for 2h, then allowed to cool to room temperature. The dark mixture was then poured into an ice/water slurry (approx. 350 mL) to precipitate out a solid. The solid was collected by vacuum filtration and dried under high vacuum overnight. The crude solid was evaporated onto celite, before being eluted through silica with 20% ethyl acetate in hexanes to give **PF1** as a white solid. (241 mg, 40%) ^{1}HNMR (500 MHz, $CDCl_3$): δ 8.01 (1H, d, *J*=7.09 Hz), 7.74 (2H, s), 7.54 - 7.62 (2H, m), 7.43 (2H, m, *J*=7.83 Hz), 7.06 (1H, d, *J*=6.85 Hz), 6.86 (2H, m, *J*=7.58 Hz), 1.33 (24H, s).

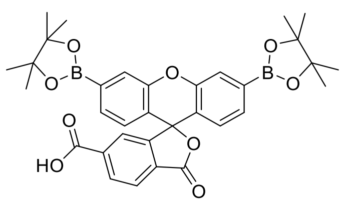

Carboxy-PF1 was prepared as per previous protocols.[1] Diiodocarboxyfluoran[3] (352 mg, 0.59 mmol), Bis(pinacolato)diboron (589 mg, 2.3 mmol), potassium acetate (500 mg, 5.9 mmol) and Pd(dppf)Cl_2 (145 mg, 5.3 mmol) were dried under high vacuum overnight. The reaction vessel was sealed, dry DMF (5 mL) was added and argon gas bubbled through. The reaction mixture was then stirred at 80 °C for 12 h, then concentrated under vacuum. The resultant crude mixture was evaporated onto celite and eluted through silica using 1% acetic acid in ethyl acetate to give carboxy-PF1 as a light brown solid. (85 mg, 24%) ^{1}HNMR (500 MHz, $CDCl_3$): δ 8.28 (1H, d, *J*=7.34 Hz), 8.10 (1H, d, *J*=7.58 Hz), 7.75 (2H, s), 7.72 (1H, s), 7.43 (2H, d, *J*=7.83 Hz), 6.82 (2H, d, *J*=7.83 Hz), 1.35 (24H, s).

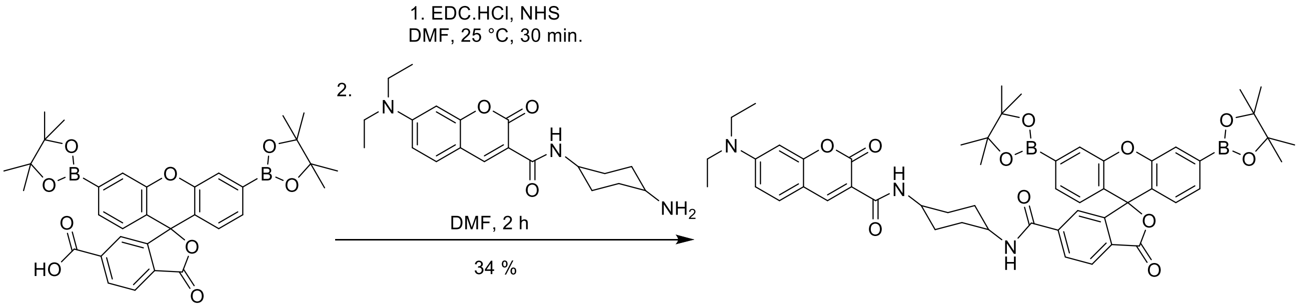

RPF1 was synthesised by an alternate route to literature.[4] Carboxy-PF1 (30 mg, 0.05 mmol), N-hydroxysuccinimide (7 mg, 0.055 mmol) and ethylcarbodiimide hydrochloride (19 mg, 0.1 mmol) were dissolved in dry DMF (3 mL) and stirred for 30 min. Coumarin linker derivative[4] was dissolved in dry DMF (3 mL) and added dropwise to reaction mixture over 10 min. The solution was stirred for another 2 h before pouring the reaction mixture into ice/water slurry (approx. 50 mL) to precipitate a solid. The solid was collected, dissolved in dichloromethane, the solution dried over sodium sulphate and then concentrated onto celite under vacuum. This was then eluted through silica using ethyl acetate/petroleum ether (2:1) to give RPF1 as a bright yellow solid (16 mg, 34%). ^{1}HNMR (500 MHz, $CDCl_3$): δ 8.71 (1H, d, *J*=7.83 Hz), 8.67 (1H, s), 8.10 (1H, d, *J*=7.83 Hz), 8.03 (1H, d, *J*=8.31 Hz), 7.77 (2H, s), 7.46 (2H, m, *J*=7.83 Hz), 7.43 (1H, d, *J*=9.05 Hz), 7.32 (1H, s), 6.85 (2H, d, *J*=7.83 Hz), 6.62-6.68 (1H, m), 6.51 (1H, s), 5.84 (1H, d, *J*=7.58 Hz) 3.93 (2H, br. s.), 3.46 (4H, q, *J*=7.17 Hz), 2.04-2.17 (4H, m), 1.42-1.49 (4H, m), 1.37 (24H, s), 1.27 (6H, m).

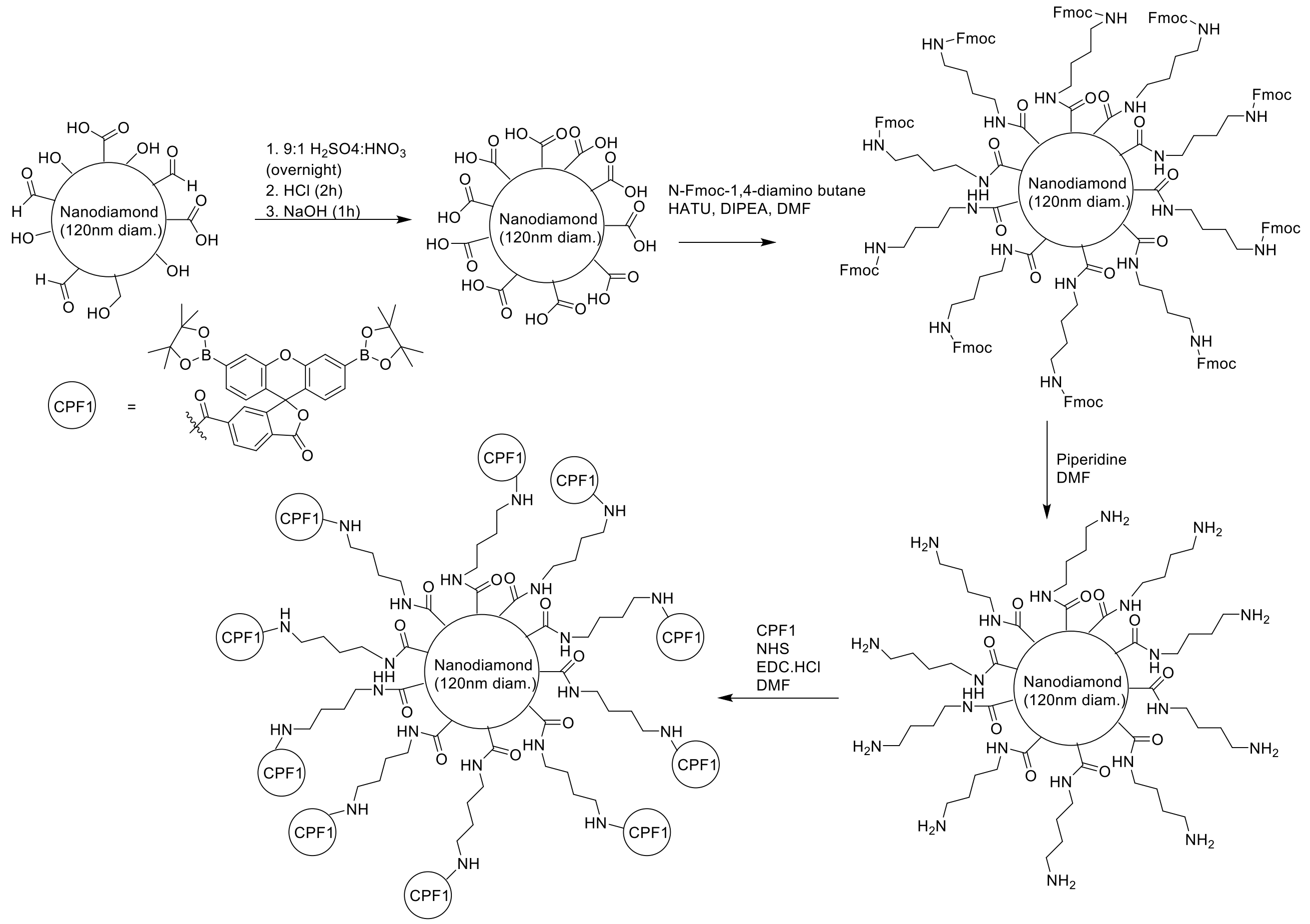

Scheme S1. Synthesis of PNS via Fmoc-protected 1,4-diaminobutane.

2. Supplementary Figures

2.1. Brightness comparison of PNS, PF1 and RPF1

The photostability of PNS, PF1 and RPF1 in manuscript **Figure 2B** are shown as normalised values from the same excitation power. However, after reacting with 1 mM H_2O_2 for 3 h, PF1 was 10x brighter than RPF1 at this initial intensity. In order to directly compare PF1 and RPF1 based on fluorescent intensity, the excitation power used for PF1 was reduced to 3% its initial value, such that the initial fluorescent intensity of PF1 and RPF1 were within the same order of magnitude. Consequently, the observed photobleaching shown by PF1 was less dramatic over 175 s than for RPF1 (**Figure 1**). This suggests that the brightness of PF1 gives an excellent advantage over RPF1, as a fraction of the excitation power is required to give a similar emission intensity, thus reducing the negative effects of photobleaching. By comparison, the nanodiamond exhibited a lower overall fluorescent intensity, however with no effects of photobleaching. This issue of brightness verses photostability was explored more fully in a previous publication [5].

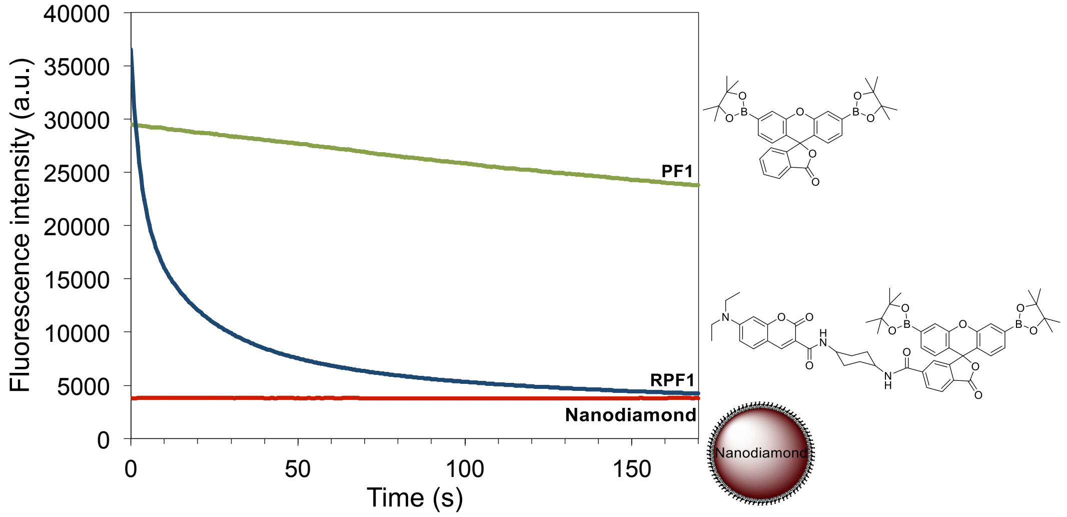

Figure S1. Photobleaching of PF1 and RPF1 with modified excitation power for similar initial fluorescence intensity (NV nanodiamond fluorescence is shown here for comparison). Fluorescence was collected from excitation and emission wavelengths of 485 nm, 520 nm (PF1); 400 nm, 520 nm (RPF1); and 560 nm, 700 nm (NV nanodiamond). Excitation intensity was 12 W.cm^{-2} (RPF1 and Nanodiamond) or 0.36 W.cm^{-2} (PF1) and emission was normalised to the initial value for each sample. All materials were dispersed in deionised water in a glass capillary with a 50 × 50 μm cross section and an image was recorded every 0.5 s for 170s.

2.2. Bio-photostability of PNS compared to PF1 and RPF1

As discussed in the manuscript; PNS, PF1, and RPF1 were incubated with pro-inflammatory M1 polarised macrophage cells for 30 min, and then imaged by confocal microscopy. A 25% reduction in fluorescent intensity was observed for PF1, and a 10% reduction observed for RPF1. The fluorescent intensity of the NV nanodiamond component of PNS remained unchanged throughout the course of imaging (see manuscript Figure 3A). Furthermore, tracking of PNS via the NV nanodiamond emission did not photobleach carboxy-PF1, the H_2O_2 sensing element of PNS (see manuscript Figure 3B).

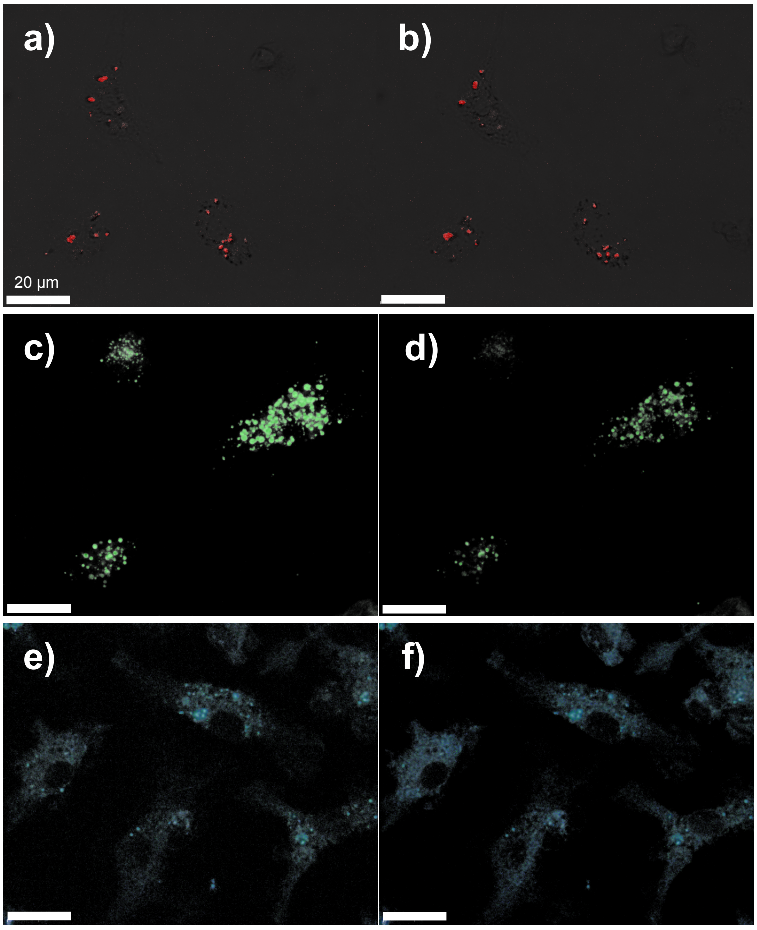

Figure S2. Confocal z-stack images (40x) of macrophages imaged five times over a 30-minute period. Photobleaching was not seen for the NV nanodiamond emission of PNS from before (A) to after (B). However, bleaching was observed for organic fluorophores PF1 (C) and (D), and RPF1 (E) and (F) respectively. Scale bars are 20 µm.

2.3. Detection of H_2O_2 as an exogenous stimulus

H_2O_2 (100 μM) was added to PNS during the macrophage polarisation process as discussed in the manuscript, and the cells imaged by confocal microscopy upon completion of polarisation. A 50% higher ratio of green/red fluorescence was observed as compared to untreated cells.

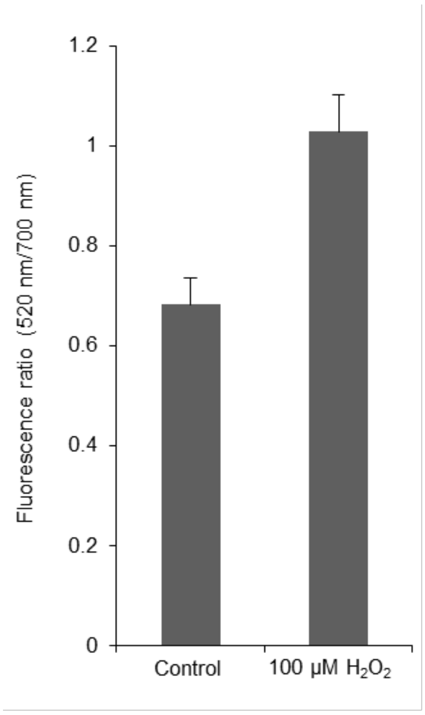

Figure S3. Increase of green (520 nm)/red (700 nm) fluorescence ratio (excitation 476 nm and 560 nm) upon addition of 100 μM H_2O_2 to PNS in macrophages ($p = 0.0410$) as compared to an untreated control. Data was collected by confocal microscopy 18 h after stimulus and was averaged from 4 locations for each condition. Error bars represent the standard error of the mean.

2.4. PNS in Macrophages after 7 days

PNS was added to the culture media on day 2 of the macrophage differentiation process, as discussed in the manuscript. After completion of differentiation and polarisation the cells were imaged by confocal microscopy and showed typical macrophage cell morphology, indicating that PNS does not affect the growth process. PNS was observed to be within some of the cells as expected, although some of the larger PNS particles were not. This could be due to exocytosis of diamond particles as has been shown in other studies.[6]

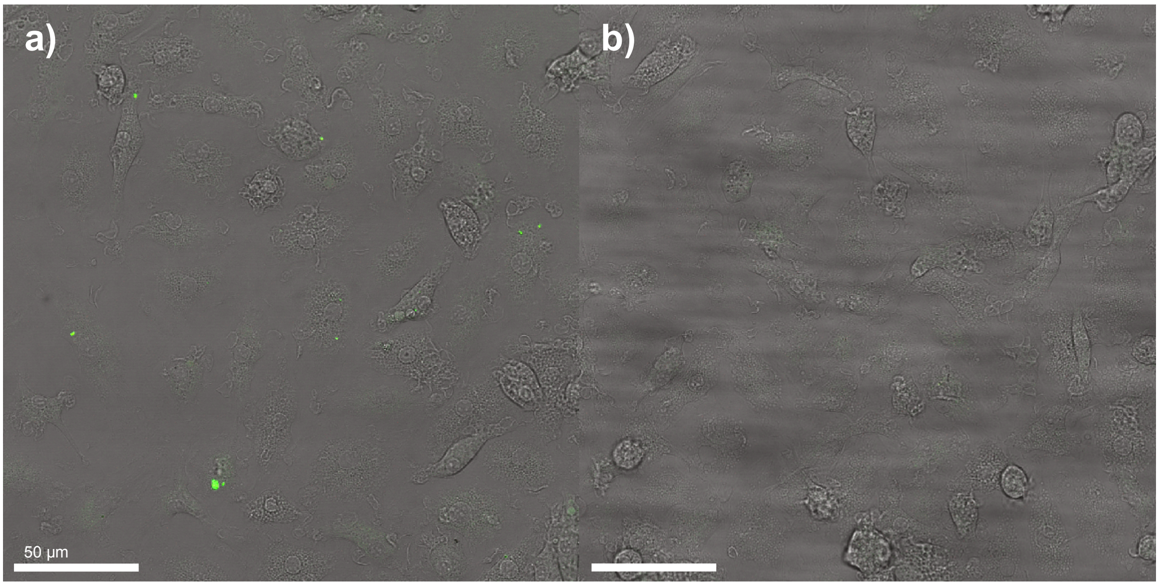

Figure S4. 40x Confocal images of macrophages after 7 days of co-incubation with PNS during differentiation and polarisation. Overlay of green (520 nm), red (700 nm) and brightfield channels. Note that PNS red fluorescence is masked by the PNS green fluorescence, which is significantly more intense. (A) Cells incubated in the presence of PNS. (B) Cells incubated without PNS. Scale bars represent 50 µm.

2.5. Fourier-Transform Infrared Spectroscopy

Fourier Transform Infrared Spectroscopy (FTIR) was performed on a Perkin Elmer S400 Infrared spectrometer in Universal ATR mode. Solid samples of PNS (blue), acid functionalised NV nanodiamond (black, product of step 1 in Scheme S1), and the amino functionalised intermediate (red, product of step 3 in Scheme S1) were used for comparison. Attachment of diamino butane linker was confirmed by the presence of characteristic C-H modes, and loss of the O-H stretch. Coupling of carboxy-PF1 was confirmed by the presence of the boronate stretching mode.

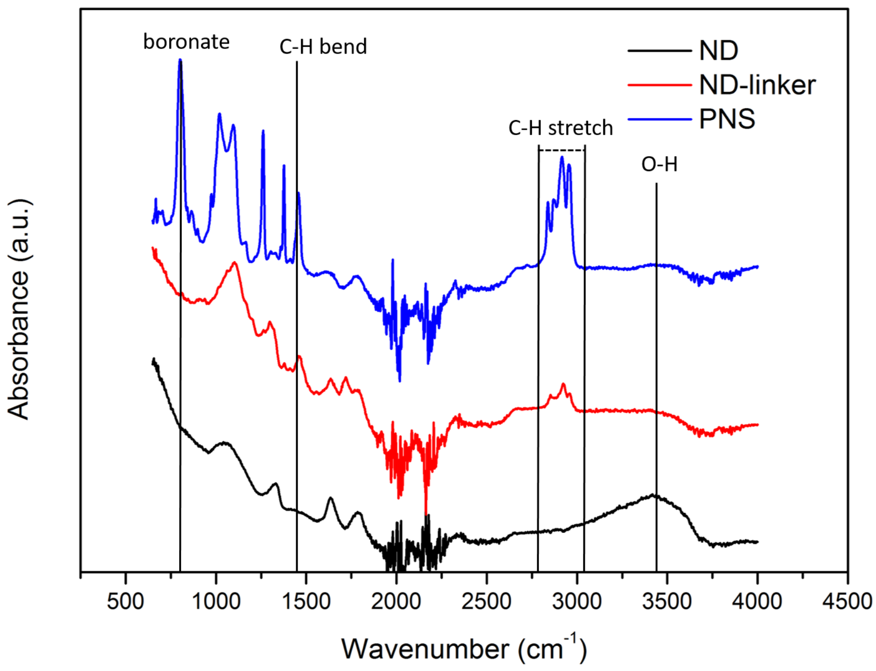

Figure S5. FTIR spectra for the acid functionalised NV nanodiamonds (black), NV nanodiamond-butane linker intermediate (red), and PNS (blue). The black vertical lines label key stretching modes as discussed in the manuscript.

3. References

1 Purdey, M. S. *et al.* Boronate probes for the detection of hydrogen peroxide release from human spermatozoa. *Free Radical Biol. Med.* **81**, 69-76, doi:10.1016/j.freeradbiomed.2015.01.015 (2015).

2 Chang, M. C. Y., Pralle, A., Isacoff, E. Y. & Chang, C. J. A selective, cell-permeable optical probe for hydrogen peroxide in living cells. *J. Am. Chem. Soc.* **126**, 15392-15393 (2004).

3 Srikun, D., Albers, A. E. & Chang, C. J. A dendrimer-based platform for simultaneous dual fluorescence imaging of hydrogen peroxide and pH gradients produced in living cells. *Chem. Sci.* **2**, 1156-1165, doi:10.1039/c1sc00064k (2011).

4 Albers, A. E., Okreglak, V. S. & Chang, C. J. A FRET-based approach to ratiometric fluorescence detection of hydrogen peroxide. *J. Am. Chem. Soc.* **128**, 9640-9641 (2006).

5 Reineck, P. *et al.* Brightness and photostability of emerging red and near-IR fluorescent nanomaterials for bioimaging. *Adv. Opt. Mater.* **4**, 1549-1557, doi:10.1002/adom.201600212 (2016).

6 Prabhakar, N. *et al.* Intracellular trafficking of fluorescent nanodiamonds and regulation of their cellular toxicity. *ACS Omega* **2**, 2689-2693, doi:10.1021/acsomega.7b00339 (2017).
